# Whole Blood Volume-Based Absolute Quantification of HTLV-1 Proviral Load: A Comparative Method Evaluation Study

**DOI:** 10.3390/v18050580

**Published:** 2026-05-21

**Authors:** Gabriel O. Franco, Andreas Stocker, Eduardo M. Netto, Heliene Pereira, Carlos Brites

**Affiliations:** 1 Laboratório de Pesquisa em Infectologia (LAPI), Complexo Hospitalar Universitário Professor Edgard Santos, Salvador 40110-060, Brazil; gabriel.osf22@gmail.com (G.O.F.); astocker@ufba.br (A.S.); nettoeduardom@hotmail.com (E.M.N.); helienepereira@outlook.com (H.P.); 2 Programa de Pós-Graduação em Medicina e Saúde (PPgMS), Universidade Federal da Bahia (UFBA), Salvador 40110-060, Brazil; 3 Centro de Formação em Biologia Molecular Charles Merieux (CFBMCM), Professor Edgard Santos University Hospital Complex, Salvador 40110-060, Brazil

**Keywords:** HTLV-1, proviral load, qPCR, absolute quantification, methodological standardization

## Abstract

The proviral load of human T-cell lymphotropic virus type 1 (HTLV-1) is an important biomarker associated with the monitoring and risk stratification of adult T-cell leukemia/lymphoma (ATL) and HTLV-1-associated myelopathy/tropical spastic paraparesis (HAM/TSP). However, the lack of standardized quantification methods limits its broader application. This study evaluated a novel absolute quantification approach based on whole blood volume and compared its performance with established protocols. A total of 66 HTLV-1-infected individuals were analyzed using six qPCR-based methodologies, including volumetric quantification (copies/µL) by absolute quantification and the Tamegão-Lopes method, as well as normalization per 1000 cells (whole blood, buffy coat, PBMCs, and CD4+ T cells). Association and agreement were assessed using Pearson’s correlation, Bland–Altman analysis, concordance correlation coefficients (CCCs), and Deming regression. Absolute quantification showed strong correlation with both the Tamegão-Lopes method and CD4+-based quantification (*r* = 0.93 and 0.84, respectively; *p* < 0.001) and high agreement (CCC = 0.866 and 0.811, respectively), with modest systematic bias (−0.273 log_10_ copies/µL and 0.115 log_10_ copies/10^3^ cells, respectively). Leukocyte-normalized methods showed greater discrepancies, likely due to dilution by uninfected cells. These findings show that quantification based on total blood volume is a simplified, operationally feasible alternative for assessing HTLV-1 proviral load.

## 1. Introduction

The human T-cell lymphotropic virus type 1 (HTLV-1) was the first identified human retrovirus and was isolated from lymphocytes of a patient with cutaneous lymphoma [1]. HTLV-1 has a wide global distribution, and current estimates suggest that between 5 and 10 million people worldwide are living with HTLV-1 infection. Most (around 90%) of these individuals remain asymptomatic throughout their life, which complicates screening and epidemiological surveillance. Brazil is the South American country with the highest HTLV-1 prevalence [2,3,4].

HTLV-1 infection is characterized by a chronic course and is associated with severe clinical manifestations, such as adult T-cell leukemia/lymphoma (ATL) and HTLV-1-associated myelopathy/tropical spastic paraparesis (HAM/TSP), as well as inflammatory conditions such as alveolitis, polymyositis, arthritis, and uveitis [5,6]. Following infection, viral RNA is converted into double-stranded DNA by the enzyme reverse transcriptase, and the resulting proviral DNA is integrated into the host cell genome, preferentially in CD4+ T lymphocytes [7,8,9]. Quantification of HTLV-1 proviral load (PVL) is an important biomarker associated with the risk of development and the rate of progression of ATL and HAM/TSP [10,11,12,13].

Despite its clinical relevance, PVL quantification methods still require standardization, and the wide variation in quantification methods limits methodological harmonization. Currently, there is substantial heterogeneity among real-time PCR (qPCR) protocols used across different centers. These variations include the choice of the appropriate biological matrix, which hinders protocol standardization and compromises comparability between studies [14,15,16,17].

One of the main obstacles for protocol standardization is the heterogeneity of normalization strategies and sample processing: many protocols use cellular fractions (buffy coat, peripheral blood mononuclear cells (PBMCs), or CD4+ cells), which introduce additional pre-analytical variability due to the handling of these materials [14,18]. Some protocols differ in how results are expressed, either as absolute concentration based on blood volume (copies/µL) or as PVL proportional to the number of cells (copies/1000 or 10^6^ leukocytes or lymphocytes), requiring normalization based on a reference gene (albumin) [16]. Other protocols also report normalization based on total leukocyte counts, highlighting the broad methodological diversity currently in use [19].

Implementing suitable methodologies for PVL quantification remains challenging in laboratory settings, making it essential to validate strategies that combine high accuracy, reproducibility, and operational feasibility. We proposed that a simplified whole blood-based approach, developed in our laboratory, could be a suitable alternative to established volumetric and cell-normalized methods for HTLV-1 PVL quantification. This study evaluated whole blood volume-based absolute quantification (WBV copies/µL of blood), based on the direct conversion of Ct values through a standard curve independent of hematological parameters or reference genes, in comparison with established volumetric and cellular protocols.

## 2. Materials and Methods

### 2.1. Study Design

This is a cross-sectional study conducted at the Complexo Hospitalar Universitário Professor Edgard Santos (COM-HUPES), a university hospital and referral center for the care of individuals with HTLV infection, located in Salvador, Bahia, Brazil. The study included a convenience sample of 53 participants over 18 years of age with HTLV-1 infection who were followed at COM-HUPES.

Participants were included regardless of antiretroviral therapy exposure status, since the primary objective of this study was to compare qPCR quantification methodologies rather than evaluate biological determinants of proviral load variation. Therefore, all biological samples obtained from each participant were analyzed using all quantification approaches, enabling direct paired comparisons between methods.

### 2.2. Ethical Aspects

This study is part of a research project that evaluated the effectiveness of antiretroviral therapy for the treatment of HTLV-1 infection, approved by the Research Ethics Committee of the Climério de Oliveira Maternity (UFBA), number 4,643,372. All participants provided written informed consent.

### 2.3. Sample Processing

Blood samples were collected to measure HTLV-1 PVL in whole blood, buffy coat, PBMCs, and CD4+ cells. After collection, 1 mL aliquots of whole blood were prepared and distributed into cryovials. Complete blood count was performed for all samples. For buffy coat isolation, samples in EDTA tubes were centrifuged at 2000× *g* for 10 min, followed by aspiration of the leukocyte layer, which was transferred into cryovials. All aliquots were stored in freezers at −80 °C.

PBMCs were isolated from whole-blood samples using a Ficoll-Paque density gradient (1.077 g/mL) [20]. After two washing steps with 1× PBS, the cells were resuspended in 1 mL of fetal bovine serum containing 10% dimethyl sulfoxide (DMSO) and cryopreserved at −80 °C.

CD4+ T-cell isolation was performed by negative selection directly from whole blood using the EasySep™ Direct Human CD4+ T Cell Isolation Kit (Stemcell Technologies, Seattle, WA, USA) in conjunction with the EasySep™ Magnet (Stemcell Technologies, Seattle, WA, USA). According to the manufacturer’s specifications, fresh samples collected in heparin tubes were used and processed immediately after collection to ensure cellular integrity. Initially, 1.5 mL of whole blood was transferred to a 5 mL polystyrene tube, to which 75 µL of Isolation Cocktail and 75 µL of RapidSpheres™ (STEMCELL Technologies Inc., Vancouver, BC, Canada) were added. After a 5 min incubation at room temperature, the volume was adjusted to 2.5 mL with 1× PBS. The tube was placed in the magnet for 5 min to immobilize unwanted cells, while the supernatant containing enriched CD4+ T cells was carefully transferred to a new tube for an additional washing step. The isolated cells were then centrifuged at 200× *g* for 10 min; the supernatant was discarded, and the resulting cell pellet was immediately stored at −80 °C for subsequent DNA extraction.

### 2.4. Sample Thawing

Whole blood, buffy coat, and CD4+ cell aliquots were thawed at room temperature (20–25 °C). For resuspension of the CD4+ cell pellet, 200 µL of 1× PBS was added to an Eppendorf tube, followed by the extraction step.

For thawing of the peripheral blood mononuclear cells (PBMCs), an optimized protocol was used to remove DMSO and preserve cell viability. The cryovial containing the sample and RPMI medium were prewarmed in a 37 °C water bath. Subsequently, RPMI was added to the cell suspension (at a 1:1 ratio). The mixture was transferred to a Falcon-type tube containing 8 mL of RPMI supplemented with 10% fetal bovine serum (FBS) and centrifuged at 2000× *g* for 10 min. The supernatant was discarded, and the resulting pellet was resuspended in 600 µL of 1× PBS, for DNA extraction.

### 2.5. Nucleic Acid Extraction

Genomic DNA extraction was performed in batches, under the same experimental conditions, using the PureLink™ Genomic DNA Mini Kit (Invitrogen, Carlsbad, CA, USA). The protocol was executed according to the manufacturer’s instructions. For each extraction, an initial volume of 200 µL of each sample was used, resulting in a final elution volume of 50 µL. The eluates were immediately stored in a freezer at −80 °C for further analysis.

### 2.6. qPCR Protocol

Proviral load determination was performed by qPCR, based on the protocol previously developed and published by Tamegão-Lopes et al. [19]. We used the TaqMan^®^ system on the CFX96™ Real-Time PCR Detection System (Bio-Rad Laboratories, Hercules, CA, USA), with the following cycling conditions: 2 min at 94 °C, followed by 45 cycles of 20 s at 94 °C and 40 s at 60 °C. The assay was designed to amplify two targets: a region of the HTLV-1 pol gene and the human albumin (ALB) gene as an endogenous control (reference gene). The primers and probes were designed and synthesized by Thermo Fisher Scientific (Waltham, MA, USA) using previously published sequences (Table 1).

In this study, six different methodological approaches were used to quantify HTLV-1 proviral load (PVL). In addition to the molecular assay, calculation and normalization strategies were compared according to the following metrics: (I) WBV (copies/µL of blood), a protocol developed and standardized in our laboratory; (II) the quantification proposed by Tamegão-Lopes (QTL); (III) PVL normalized per 1000 leukocytes, applied to whole blood (WHL copies/10^−3^ cells), buffy coat, and PBMC fractions (PBMC copies/10^−3^ cells); and (IV) PVL per 1000 lymphocytes, using isolated CD4+ cells (CD4+ copies/10^−3^ cells). Although all methods were based on the same molecular assay, they differed in the normalization denominator used and the biological matrix analyzed.

### 2.7. Standard Curve

Standard curves were established for both HTLV-1 and ALB targets for PVL quantification and normalization. The curves were generated from serial dilutions of purified and previously quantified amplicons, ranging from 10^−1^ to 10^−14^. After evaluating linearity, the dynamic range used to construct the standard curve was defined between 10^−3^ and 10^−10^, corresponding to the range with the best linear performance and amplification stability [14,21,22]. The initial concentration of genetic material was determined by fluorometry using the Qubit™ system with the HS DNA kit (Invitrogen, Carlsbad, CA, USA), for copy number calculation based on the molecular mass of the amplicons. Assay performance was initially validated using purified amplicons quantified by fluorometry, ensuring linearity and amplification efficiency prior to clinical sample analysis. A detailed validation of the standard curve, including inhibition assessment, exclusion of the stochastic limit, and the regression parameters, is described in Appendix B.

The HTLV-1 standard curve showed an efficiency of 94.5%, with a coefficient of determination (R^2^) of 0.99, slope of −3.46, and a limit of detection of 6 copies/µL. For the albumin (ALB) gene, an efficiency of 97.6%, R^2^ of 0.99, slope of −3.38, and a limit of detection of 9 copies/µL were obtained. These parameters fall within the recommended criteria for real-time PCR assays, according to MIQE guidelines [23].

Standard curve reactions were performed in duplicate to ensure quantification accuracy. Clinical samples were analyzed in singlet, consistent with the study’s objective of comparing different methodological approaches under standardized experimental conditions, applied equally to all samples. Analytical quality was monitored through standard curve efficiency and amplification performance of the endogenous gene (ALB).

A correction factor was established to ensure result reliability and compensate for systematic variations associated with the extraction process. Six samples were initially extracted; subsequently, 20 µL of each eluate was diluted in 180 µL of saline solution (0.9% NaCl) to simulate a diluted matrix, prior to a new extraction step. qPCR analysis showed a mean difference of ΔCt = 2.01 between the original and re-extracted eluates. Considering the exponential nature of qPCR, this difference corresponds to approximately a four-fold variation in copy number estimation. This ΔCt value was incorporated as a correction factor in the final equations for all HTLV-1 PVL calculations and was added to the Ct values obtained from clinical samples prior to conversion into copy numbers. The application of this correction factor was standardized to reduce variability associated with the extraction process.

Sensitivity analyses using different correction-factor scenarios were additionally performed to evaluate the robustness of the proposed quantification approach (Appendix B).

### 2.8. Determination of Proviral Load

WBV (copies/µL of blood) was determined through amplification of the specific target (HTLV-1), independently of normalization by an endogenous control (ALB). The main analytical tool was the previously established reference standard curve, which enabled the definition of a linear relationship between fluorescence and DNA quantity.

〖PVL = 10〗^((a × (Ct + CF) + b) (1)

Equation (1) was used to convert Ct values into copy numbers, where “a” represents the slope of the curve, “CF” the correction factor obtained, and “b” the intercept. Final values were expressed as HTLV-1 copies/µL.

The determination of PVL proposed by Tamegão-Lopes, et al. [19]. is initially based on a ratio derived from the quantitative relationship between the Ct of the target (HTLV-1) and the endogenous control (ALB). Subsequently, these values were multiplied by the patient’s total leukocyte count and by a factor of “2,” corresponding to the diploid nature of the cellular genome. The PVL results were expressed as HTLV-1 copies/mm^3^.

(PVL = 2^(−Ct HTLV1)/2^(−CT Albumin) × Leukocytes × 2(2)

Equation (2) is the final calculation proposed by Tamegão-Lopes et al. [19].

The measurement of PVL normalized by cell count (copies/1000 cells) was also evaluated [24]. These protocols estimate the proportion of HTLV-1 copies per 1000 leukocytes (whole blood, buffy coat, and PBMCs) or per CD4+ lymphocytes. To determine the ratio between the number of HTLV-1 copies and the number of cells, the calculation is based on quantification of the albumin gene, with the number of copies of this gene divided by two to represent the cellular genomic equivalent [25,26]. The absolute copy numbers of the HTLV-1 and albumin (ALB) gene were determined using the standard curve.

(PVL = ((HTLV1 Copies))/(((ALB Copies)⁄2)) × 1000)(3)

Equation (3) was the final equation used to determine PVL per 1000 cells.

### 2.9. Handling of Missing Data

**Figure 1:**
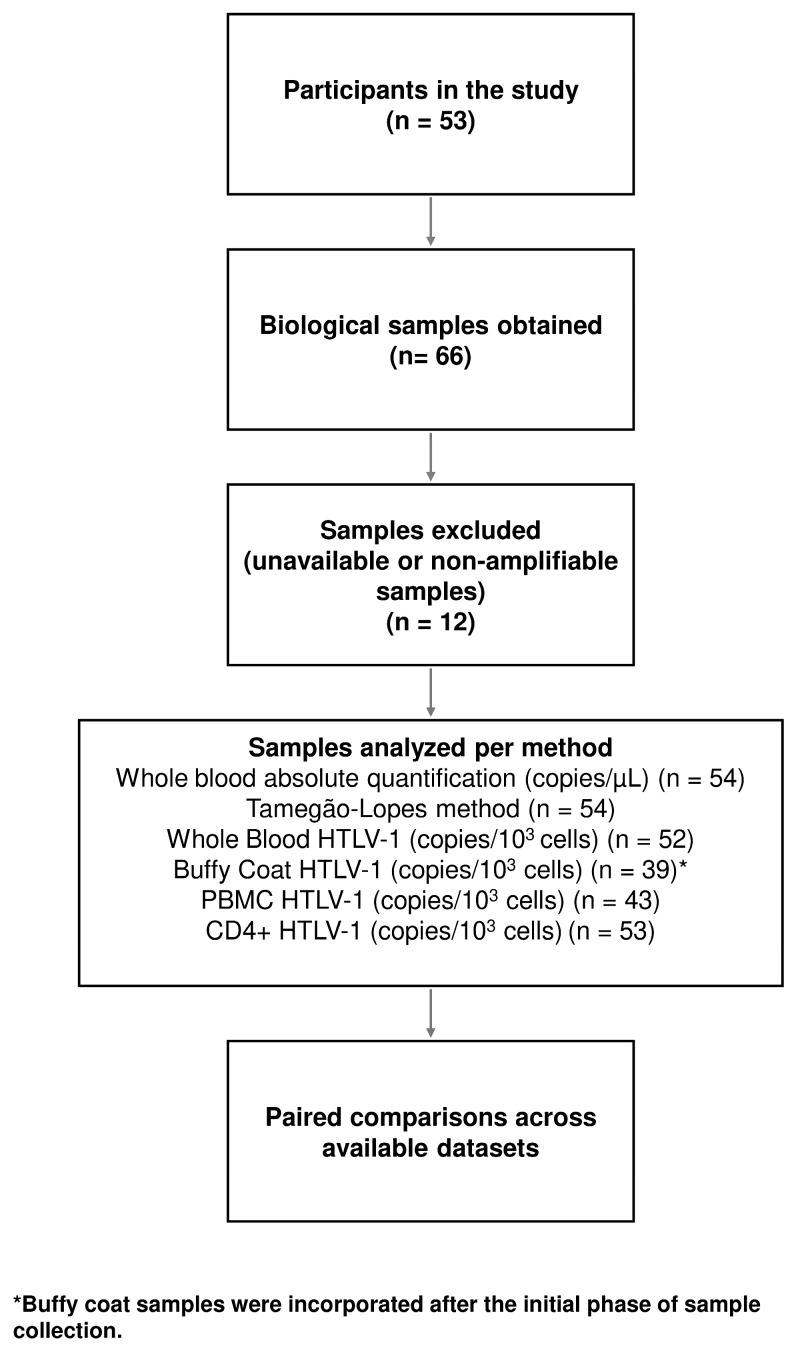
Flowchart of the selection and processing of biological samples. The diagram illustrates the path from the inclusion of participants (N = 53) to the final paired analyses. It details the exclusions of unavailable or non-amplifiable samples and the final quantity submitted to each of the six HTLV-1 proviral load quantification protocols.

Samples were included in each analysis according to the availability and quality of biological material for the corresponding qPCR assay. Buffy coat samples were incorporated after the initial phase of sample collection and were therefore unavailable for a subset of participants. Additional missing data were related to unsuccessful CD4+ cell isolation, insufficient sample volume, or absence of HTLV-1 amplification in the analyzed specimens. No exclusions were made based on proviral load levels or predefined cell-count thresholds; all analyses were conducted using the available paired samples for each methodological comparison. Figure 1 shows the sample selection and progression flow.

### 2.10. Statistical Analysis

PVL variables were log_10_-transformed and analyzed to assess association and agreement between measurements. Means were compared using the paired Student’s *t*-test, and linear association between the variables was evaluated using Pearson’s correlation coefficient (*r*). Agreement between the measurements was assessed by Bland–Altman analysis [27], through estimation of the mean difference (bias) and the 95% limits of agreement, as well as the concordance correlation coefficient (CCC) [28]. A *p*-value < 0.05 was considered statistically significant. Statistical analyses were performed using Jamovi software, version 2.3.

## 3. Results

The comparison was structured in three sequential steps. First, whole blood volume-based absolute quantification was compared with the protocol proposed by Tamegão-Lopes et al. [19]. to validate the accuracy of our approach for quantifying proviral load in copies per microliter (µL). Subsequently, the analysis was focused on protocols normalized per 1000 cells (leukocytes and lymphocytes) across different biological matrices: whole blood, buffy coat, PBMCs, and CD4+ cells. For this comparison, the protocol using isolated CD4+ cells was adopted as the biological reference, considering the preferential tropism of HTLV-1 for CD4+ T lymphocytes, which represent the main in vivo proviral reservoir. Finally, WBV (copies/µL of blood) was compared with the protocols normalized per 1000 cells to evaluate potential interchangeability between the methods.

### 3.1. Demographic and Clinical Profile

Study participants had a mean age of 56.3 ± 9.3 years, with a predominance of females (80.3%). Most individuals were symptomatic (75.9%).

### 3.2. Validation of Volume-Based Quantification (copies/µL)

Table 2 presents all comparative analyses between the methods for quantifying proviral load by blood volume (copies/µL), comparing the performance of the quantification with the protocol proposed by Tamegão-Lopes, et al. [19]. Comparison of the means using the paired t-test showed statistically significant differences between WBV (copies/µL of blood) and QTL (*p* < 0.001), although with similar average values. WBV (copies/µL of blood) showed a lower mean (1.73 ± 0.72 log_10_) compared to the QTL protocol (2.00 ± 0.75 log_10_).

**Figure 2:**
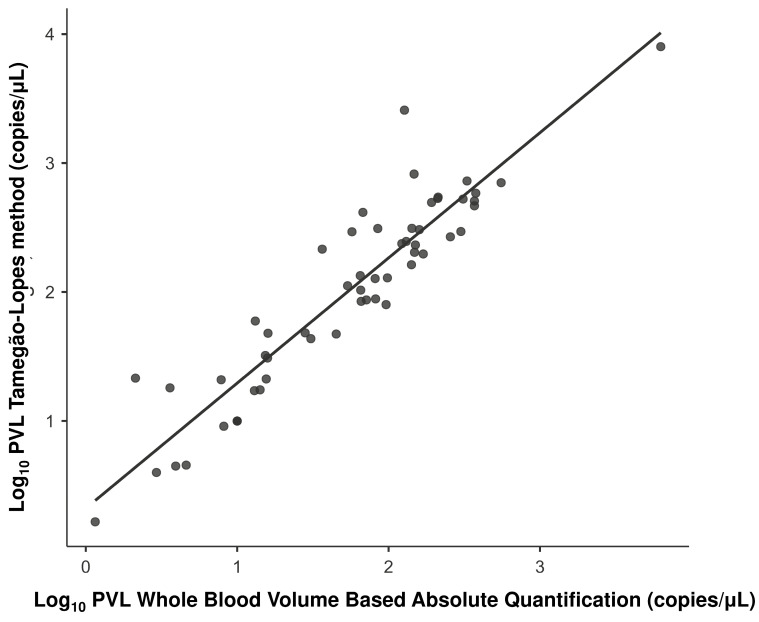
Correlation analysis between whole blood volume-based absolute quantification and the Tamegão-Lopes method.

The Pearson correlation analysis between the quantification strategies revealed strong and significant positive associations for all comparisons (Table 2; *p* < 0.001). The correlation between WBV (copies/µL of blood) and the QTL method reached the highest correlation coefficient (*r* = 0.93). Figure 2 shows the positive linearity between these two protocols and confirms the technical robustness of the two blood volume-based quantification methods.

**Figure 3:**
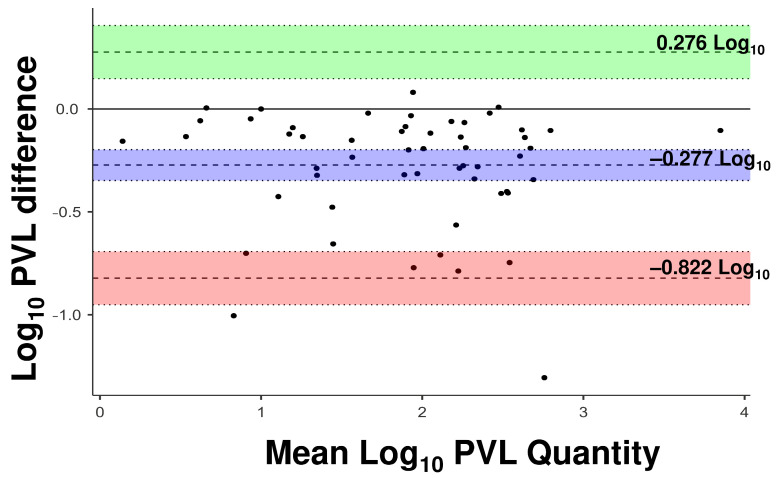
Bland–Altman plot evaluating the agreement between WBV (copies/µL of blood) and the QTL protocol. The purple shaded area indicates the mean difference (bias) with its 95% confidence interval (CI) bounded by dotted lines; the green and red shaded areas represent the upper and lower 95% limits of agreement (LoA), respectively, each with its corresponding 95% CI. Dashed lines represent the point estimates for the bias and limits of agreement.

The concordance analyses reinforced the clinical applicability of WBV (copies/µL of blood), which showed the highest concordance correlation coefficient (CCC = 0.866) with the QTL protocol (Table 2). The Bland–Altman plot for this comparison (Figure 3) revealed a mean bias of −0.273 log_10_, indicating a slight underestimation, but with narrow limits of agreement (−0.822 and 0.276) and points evenly distributed around the mean, with minimal systematic difference between the blood volume-based quantification methods.

Correlation and concordance metrics remained largely stable across sensitivity scenarios, whereas Bland–Altman bias varied according to the correction-factor magnitude (Appendix B).

### 3.3. Validation of Biological Matrices Based on Protocols Normalized per 1000 Cells

**Figure 4:**
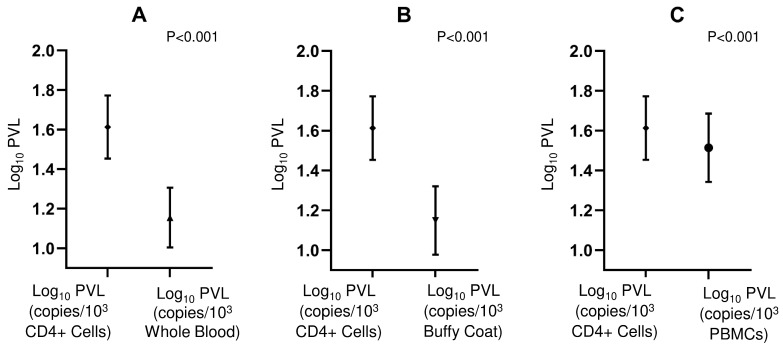
Comparison of HTLV-1 PVL quantification across different biological compartments based on the ratio of HTLV-1 copies to albumin copies per 10^3^ cells. (**A**) Comparison between Log_10_ PVL in CD4+ cells and Whole Blood; (**B**) Comparison between Log_10_ PVL in CD4+ cells and Buffy Coat; (**C**) Comparison between Log_10_ PVL in CD4+ cells and PBMCs. Statistical significance is indicated by *p*-values (*p* < 0.001) displayed in each panel.

The analysis focused on protocols normalized per 1000 cells, using the isolated CD4+ compartment as the biological reference. Comparison of the means revealed that the CD4+ compartment consistently exhibited higher HTLV-1 PVL than all other matrices analyzed (Figure 4). The largest statistical differences were observed between CD4+ and WHL (*p* < 0.001) and CD4+ and buffy coat (*p* < 0.001), whereas the closest agreement was observed with PBMC copies/10^−3^ cells (*p* < 0.001) (Table 3).

**Figure 5:**
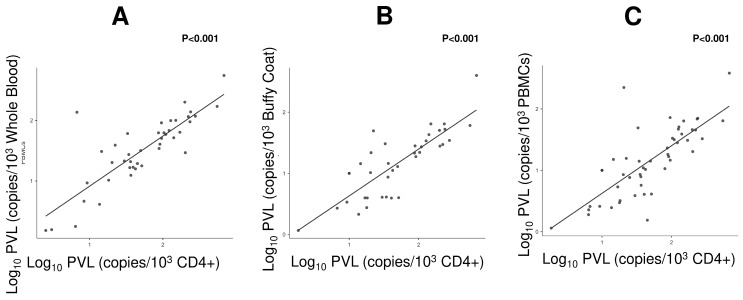
Correlation analysis between quantification per 1000 lymphocytes (CD4+) and other protocols standardized by 1000 leukocytes. (**A**): Whole blood (copies/10^−3^ cells); (**B**): buffy coat (copies/10^−3^ cells); (**C**): PBMCs (copies/10^−3^ cells).

Despite differences in mean PVL due to the cellular heterogeneity of each matrix, Pearson correlation analyses between the protocols normalized per 1000 cells revealed strong and statistically significant positive associations with the target cell compartment (CD4+) (Table 4). The highest correlation coefficient was observed between CD4+ and PBMCs (*r* = 0.83). Figure 5 illustrates this linear association between both methods normalized per 1000 cells. Although the CD4+ compartment showed the highest viral density, variations in proviral load in the other biological matrices tested (whole blood, buffy coat, and PBMCs) followed the variations observed in the biological reference.

**Figure 6:**
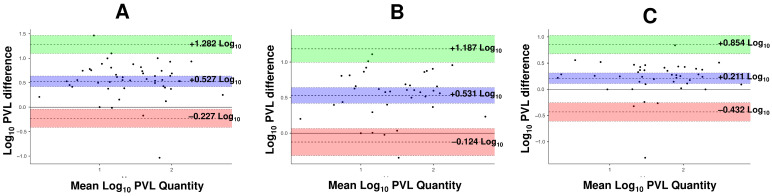
Bland–Altman plot evaluating the agreement between quantification by 10^3^ lymphocytes (CD4+) and other protocols standardized by 10^3^ leukocytes. The method using CD4+ was compared with (**A**) whole blood (copies/10^−3^ cells), (**B**) buffy coat (copies/10^−3^ cells), and (**C**) PBMCs (copies/10^−3^ cells). Differences are plotted against the mean log_10_ PVL (copies/10^3^ cells). The purple shaded area represents the 95% CI of the mean bias (dashed line); the green and red shaded areas represent the 95% CIs of the upper and lower limits of agreement, respectively.

Among the protocols normalized per 1000 cells, the protocol reporting HTLV-1 copies per 1000 lymphocytes (CD4) showed the highest concordance with PBMCs (CCC = 0.782). In contrast, WHL and buffy coat exhibited the lowest concordance (CCC = 0.520 and 0.555, respectively), with positive biases exceeding 0.500 log_10_ (Table 5). The corresponding Bland–Altman plots illustrate that most WHL and buffy coat samples lie above the zero line, indicating a tendency to underestimate proviral load when compared to the main target cell compartment (CD4+), due to the heterogeneity of the cellular population (Figure 6).

### 3.4. Interchangeability Between Methods

**Figure 7:**
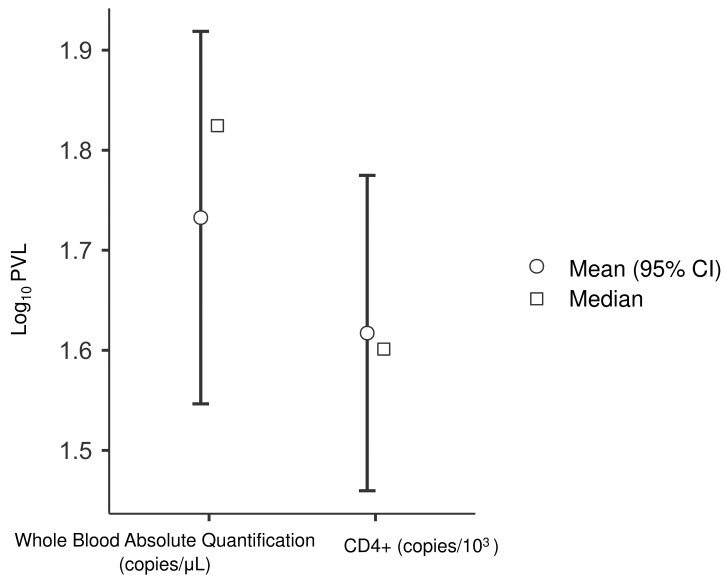
Distribution of HTLV-1 PVL compared between whole blood volume-based absolute quantification based on whole blood volume and the method based on CD4+ cells. PVL is expressed in log_10_ copies/µL for whole blood and log_10_ copies/10^3^ cells for CD4+ cells. The comparison highlights the distribution profiles between the different quantification metrics.

**Figure 8:**
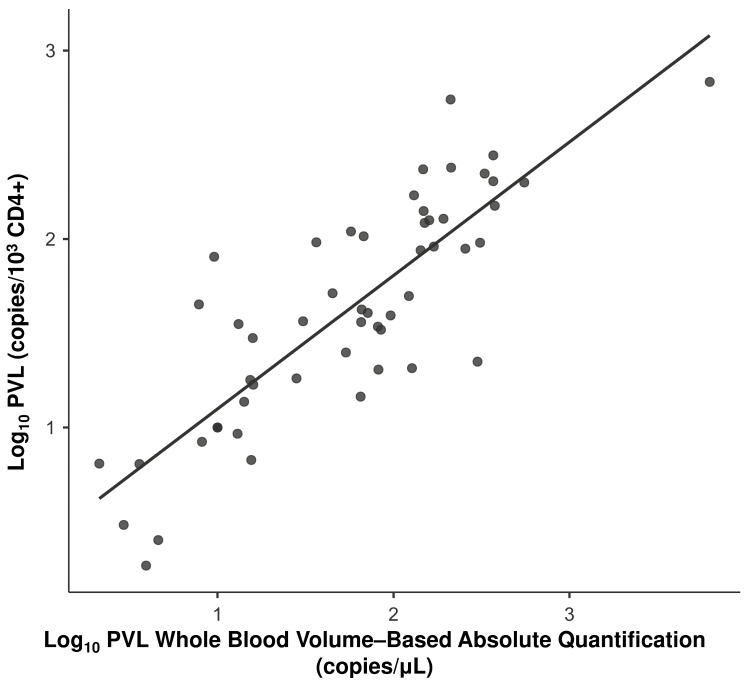
Correlation analysis between whole blood volume-based absolute quantification based on total blood volume (copies/µL) and quantification per 1000 lymphocytes (CD4+). PVL was compared between whole blood volume-based absolute quantification based on total blood volume (*x*-axis) and on isolated CD4+ cells (*y*-axis). Data are expressed in log_10_ copies/µL and log_10_ copies/10^3^ cells, respectively. The solid line represents linear regression, indicating the strong association between the two quantification metrics.

The analysis of interchangeability between protocols was used to evaluate WBV (copies/µL of blood) in comparison to the CD4+-based protocol, normalized per 1000 cells. The paired t-test indicated a statistically significant difference between the mean values (*p* < 0.031) (Table 6), and Figure 7 showed overlapping confidence intervals between the two methods. This proximity is reflected by a strong positive correlation (*r* = 0.84; *p* < 0.001) (Table 6). The clear linearity shown in Figure 8 confirms the robustness of volumetric quantification in reflecting the proviral load of the target cell compartment.

**Figure 9:**
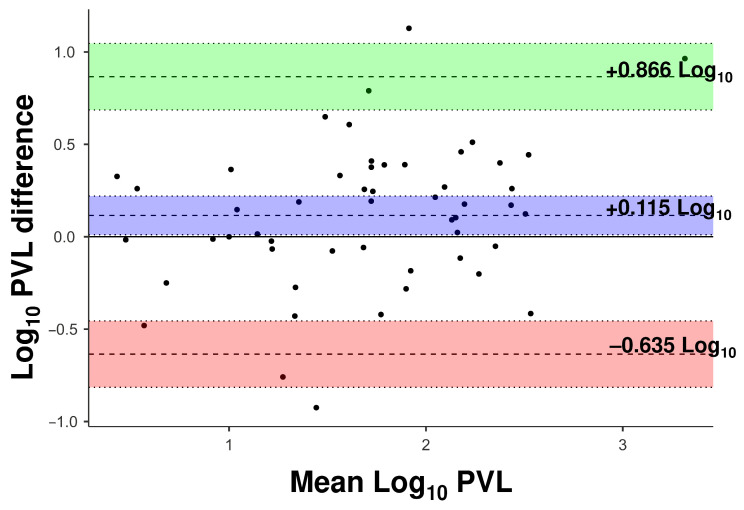
Bland–Altman plot evaluating the agreement between whole blood volume-based absolute quantification based on total blood volume and quantification per 1000 lymphocytes (CD4+). Differences are plotted against the mean log_10_ PVL (copies/µL—copies/10^3^ cells). The purple shaded area represents the 95% CI of the mean bias (dashed line); the green and red shaded areas represent the 95% CIs of the upper and lower limits of agreement, respectively. The graph illustrates the systematic agreement between the two different quantification methodologies for HTLV-1 proviral load.

Methodological robustness was further reinforced by the concordance analyses. The comparison between WBV (copies/µL of blood) and CD4+ showed a CCC of 0.811, surpassing the concordance observed for WHL (copies/10^−3^ cells) and buffy coat matrices when normalized per 1000 cells (Table 6). Bland–Altman analysis revealed the lowest systematic bias between the protocols, with a mean bias of 0.115 log_10_, narrow limits of agreement, and uniform distribution (Figure 9). These findings underscore the analytical precision and clinical feasibility of WBV (copies/µL of blood) as a viable and robust alternative to cell normalization-dependent protocols.

**Figure 10:**
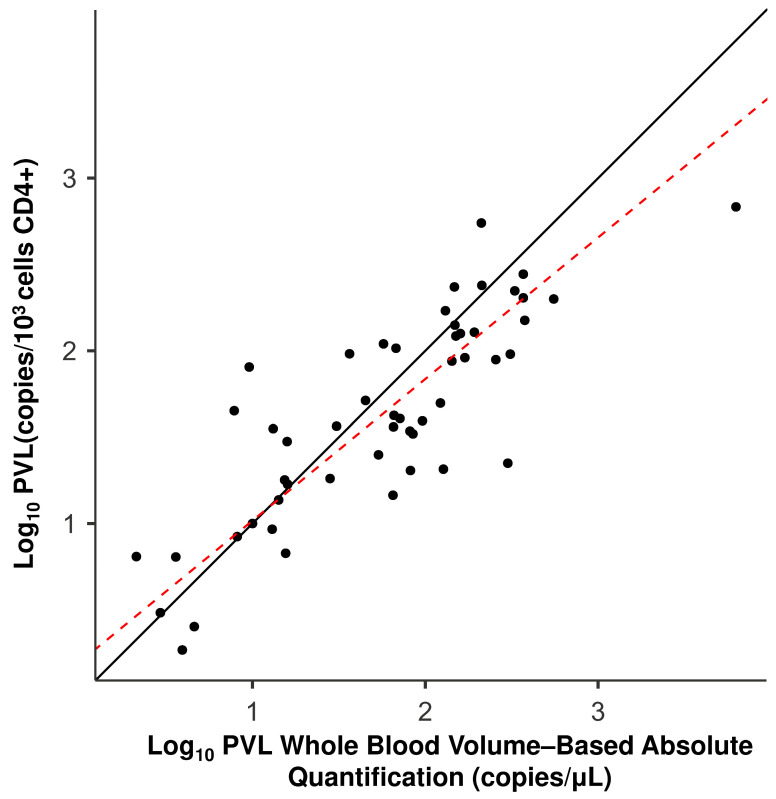
Deming regression analysis comparing volumetric quantification and CD4+ normalization. PVL data are expressed as log_10_ copies/µL (*x*-axis) and log_10_ copies/10^3^ cells (*y*-axis). The solid black line represents the identity line (y = x); the dashed red line indicates the Deming regression fit.

Deming regression analysis was used to evaluate the interchangeability between the WBV (copies/µL of blood) protocol and the protocol normalized per 1000 lymphocytes based on isolated CD4+ cells (Table 7), assuming a ratio of measurement error variances (λ) equal to 1. The scatter plot distribution showed good agreement between the methods (Figure 10).

We observed an intercept of 0.197 (95% CI: −0.082 to 0.476) and a slope of 0.820 (95% CI: 0.664 to 0.975) in the comparison between WBV (copies/µL of blood) and the CD4+ compartment. The inclusion of zero within the intercept confidence interval indicates the absence of a constant systematic error between the methods. The absence of 1.0 in the confidence interval suggests the presence of a slight proportional systematic difference, where WBV (copies/µL of blood) tends to yield slightly different values at higher proviral load ranges compared to CD4+ isolation.

## 4. Discussion

The findings of this study demonstrate a strong linear correlation and high concordance (CCC = 0.866) between whole blood volume-based absolute quantification (copies/µL) and the protocol proposed by Tamegão, et al. [19]. (*r* = 0.93). These results indicate a robust comparability between methods that express measurements using total blood volume as a denominator. The strong statistical association validates the precision of our protocol in producing consistent results when compared to previously described and validated protocols in the literature. Such findings support monitoring PVL by absolute volume as a reliable and reproducible analytical strategy. The stability of this correlation demonstrates that WBV (copies/µL of blood) is a robust methodology comparable to current standard volumetric methods.

The systematic difference observed between the QTL protocol and WBV (copies/µL of blood) (mean bias of −0.273 log_10_) may be related to the mathematical structure of the calculation used. Unlike our methodology, which uses the actual blood volume (µL) as a direct denominator, the QTL protocol is based on the relative ratio between the viral target and the endogenous gene (ALB), multiplied by the total leukocyte count and corrected by a cellular diploidy factor. This model incorporates multiple variables that can influence the final estimated value, including the relative efficiency of qPCR assays and the accuracy of hematological counts. Since each of these terms is multiplicative, small variations in any component can amplify the final estimated PVL. In contrast, the volumetric model of WBV (copies/µL of blood) reduces the number of intermediate steps and eliminates dependence on external hematological parameters.

Systematic discrepancies observed among the protocols normalized per 1000 cells become evident when compared to the isolated CD4+ compartment. The WHL (copies/10^−3^ cells) and buffy coat matrices based on protocols normalized per 1000 leukocytes exhibited the largest systematic biases (0.527 and 0.531 log_10_, respectively), which can be explained by the high cellular population heterogeneity in these matrices. As the quantification of albumin gene copies occurs in all nucleated cells [25,29], the presence of cells that do not harbor the HTLV-1 provirus, such as granulocytes, creates a mathematical dilution effect caused by the cellular composition that affects the ratio of HTLV-1 copies to albumin copies. This is because T lymphocytes are the predominant cells infected by HTLV-1 [30,31,32,33], making leukocyte-based normalization less precise in these complex matrices. In contrast, differences relative to PBMCs (0.211 log_10_), which showed the highest concordance observed among matrices compared to CD4+ (CCC = 0.782), reflect the close similarity between that cell fraction and the lymphocyte compartment.

The strong concordance between WBV (copies/µL) and CD4+ normalized per 1000 lymphocytes (CCC = 0.811), combined with the low systematic bias, indicates comparable performance between the approaches. This interchangeability is further supported by Deming regression analysis, which showed no constant systematic error (Intercept: 0.197; 95% CI: −0.082 to 0.476). However, the slope below unity (0.820; 95% CI: 0.664 to 0.975) indicates a proportional error, revealing that the relative differences between methods increase at higher proviral load ranges. Nevertheless, the magnitude of this discrepancy is limited and may not affect the performance of the WBV (copies/µL of blood) protocol for clinical application.

These findings indicate that WBV (copies/µL) represents a consistent alternative to the CD4+-based protocol. The use of whole blood has the advantage of eliminating complex pre-analytical steps that can introduce additional errors and increase the operational cost to the diagnosis for clinical monitoring.

From a laboratory and large-scale implementation perspective, WBV (copies/µL of blood) offers significant operational advantages. The volumetric method relies exclusively on quantification of the viral target (HTLV-1), eliminating the need for a reference gene (albumin) as the calculation denominator. This simplification reduces the number of reactions required per sample and decreases the consumption of primers, probes, and qPCR reagents, while also optimizing the assay execution time. The use of a previously established standard curve on a routine thermocycler allows direct conversion of Ct values into the absolute number of copies per µL of total blood, maintaining analytical traceability without reliance on additional hematological parameters.

In addition, WBV (copies/µL of blood) does not require global leukocyte counts obtained from a complete blood count. This optimizes the pre-analytical phase, as only a single tube is needed for blood collection. This feature is particularly relevant in molecular biology laboratories that do not have their own hematology analyzers, reducing fragmentation in the analytical workflow and potentially increasing process standardization and result traceability. Using total blood as the primary matrix also provides a logistical advantage over other protocols normalized per 1000 cells, which require cell fractionation (buffy coat, PBMCs, and isolated CD4+), additional processing steps, and greater technical infrastructure. Reducing these pre-analytical steps can contribute to lower technical variability and greater feasibility for implementation in routine clinical settings and epidemiological surveillance.

The small sample size is a limitation of our study. Nevertheless, it allowed a robust comparative analysis between the different protocols evaluated. From a technical standpoint, the effectiveness of WBV (copies/µL of blood) depends on a standard curve with high precision, requiring efficiencies close to 100% and minimal variation among serial dilutions—essential characteristics of well-standardized qPCR assays. The pre-analytical phase was conducted under strict standardization to minimize potential sources of variation between different samples and biological matrices. Isolation of CD4+ cells, PBMC processing, and buffy coat preparation require specialized training to reduce inter-sample variability. Finally, the simultaneous processing of four matrices per patient constituted a methodological strength of the study, ensuring direct comparability between protocols, as all PVL comparisons were performed under the same temporal and analytical conditions.

## 5. Conclusions

Whole blood volume-based absolute quantification (copies/µL of blood) is a precise, reproducible, and operationally advantageous tool for determining HTLV-1 PVL. The method showed high concordance with the CD4+-based protocol, overcoming the limitations observed in leukocyte-normalized protocols (whole blood and buffy coat), which are more susceptible to interference from cellular heterogeneity. WBV (copies/µL of blood) eliminates dependence on external hematological parameters and simplifies the laboratory workflow, reducing costs and processing time. Therefore, this protocol represents a reliable and feasible alternative for large-scale clinical monitoring, facilitating access to high-precision molecular surveillance across diverse public health settings.

## Figures and Tables

**Table 1:** Sequence of primers (HTLV-1 and ALB).

Primers/Probes	Sequences
HTLV-1 Forward	5′-GAACGCTCTAATGGCATTCTTAAAACC-3′
HTLV-1 Reverse	5′-GTGGTTGATTGTCCATAGGGCTAT-3′
HTLV-1 Probe	FAM-ACAAACCCGACCTACCC-MGB-NFQ
Albumin Forward	5′-GCTCAACTCCCTATTGCTATCACA-3′
Albumin Reverse	5′-GGGCATGACAGGTTTTGCAATATTA-3′
Albumin Probe	FAM-TTGTGGGCTGTAATCAT-MGB-NFQ

**Table 2:** Comparative analysis between WBV (copies/µL of blood) and the QTL method.

Comparison	Mean ± SD (log_10_)	*r*	*p*	Bias (log_10_)	CCC	LoA (95%)
WBV (copies/µL of blood) vs. QTL method	1.73 ± 0.72 2.00 ± 0.75	0.93	<0.001	−0.273	0.866	−0.822/0.276

Notes: *r* refers to Pearson’s correlation coefficient; *p* indicates statistical significance; bias represents the mean difference (log_10_ copies/µL) derived from Bland–Altman analysis; CCC denotes Lin’s concordance correlation coefficient (95% confidence interval); LoA indicates the 95% limits of agreement.

**Table 3:** Comparison of paired means between protocols normalized to 1000 cells.

Comparison	n	Mean Test (±SD)	95% CI	*p* Value
CD4+ vs. Whole Blood	51	1.68 ± 0.55 1.15 ± 0.56	0.42–0.63	<0.001
CD4+ vs. Buffy Coat	38	1.70 ± 0.57 1.16 ± 0.53	0.42–0.64	<0.001
CD4+ vs. PBMCs	42	1.72 ± 0.57 1.51 ± 0.57	0.11–0.31	<0.001

Notes: SD: Standard deviation.

**Table 4:** Correlation matrix between protocols for quantifying proviral load by volume and normalized per 1000 cells.

Methods	n	Correlation Coefficient (*p*)	95% CI
CD4+ vs. Whole Blood	51	0.76 *	0.61–0.85
CD4+ vs. Buffy Coat	38	0.82 *	0.67–0.97
CD4+ vs. PBMCs	42	0.83 *	0.71–0.91

(*) *p* < 0.001; Note: Data transformed using log_10_ for the application of the Pearson test.

**Table 5:** Analysis of agreement between methods for quantifying proviral load per 10^3^ cells.

Comparison	Mean Bias log_10_	Lower Limit (95%)	Upper Limit (95%)	CCC (CI 95%)
CD4+ vs. Whole Blood	0.520	−0.227	1.282	0.520 (0.365/0.647)
CD4+ vs. Buffy Coat	0.531	0.124	1.187	0.555 (0.385/0.689)
CD4+ vs. PBMCs	0.211	−0.432	0.854	0.782 (0.642/0.872)

**Table 6:** Performance of the WBV (copies/µL of blood) protocol compared to protocols normalized by 10^−3^ CD4+ cells.

Comparison	Mean ± SD (log_10_)	*p*	*r*	Bias (log_10_)	CCC (95%)	LoA (95%)
WBV (copies/µL of blood) vs. CD4+	1.73 ± 0.70 1.62 ± 0.62	0.031	0.84	0.115	0.811 (0.704/0.883)	−0.635/0.866

Note: *p* refers to the paired Student’s *t*-test; *r* refers to Pearson’s correlation coefficient; bias represents the mean difference (log_10_ copies/µL) from the Bland–Altman analysis; CCC (Lin’s concordance correlation coefficient with 95% CI); LoA (95% limits of agreement).

**Table 7:** Deming regression analysis comparing quantification protocols by volume and normalized by 1000 cells.

Comparison	Intercept (95% CI)	Slope (95% CI)
WBV (copies/µL of blood) vs. CD4+	0.197 (−0.082/0.476)	0.820 (0.664/0.975)

**Figure A1:**
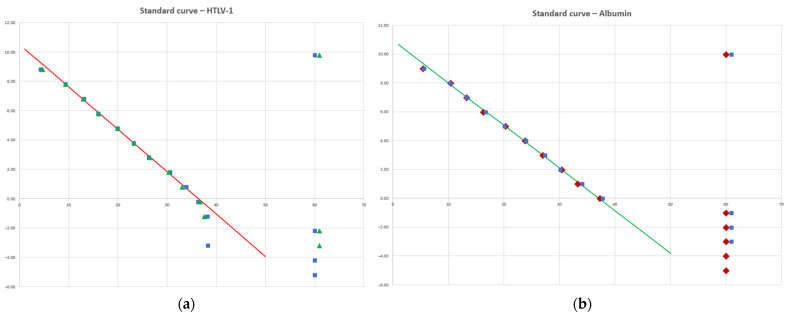
qPCR standard curves for whole blood volume-based absolute quantification. (**a**) Standard curve for the HTLV-1 pol gene, showing a linear dynamic range at serial dilutions. (**b**) Standard curve for the endogenous human albumin (ALB) control, used for cell normalization. All standards were analyzed in duplicate in different experimental assays, with each assay indicated by distinct symbols. Outliers plotted in the rightmost section of the graphs represent extreme dilutions that did not amplify within the quantifiable range; these values were assigned artificial Ct 60 or Ct 61 values to indicate non-amplification, distinguishing each specific experimental assay. The *y*-axis represents the logarithm of the DNA copy number and the *x*-axis represents the cycle threshold (Ct) values.

## Data Availability

The data that support the findings of this study are available from the corresponding author upon reasonable request. The data are not publicly available due to ethical and privacy restrictions.

## Abbreviations

The following abbreviations are used in this manuscript:

ALB	Human Albumin Gene (Endogenous Control)
ART	Antiretroviral Therapy
ATL	Adult T-cell Leukemia/Lymphoma
CCC	Concordance Correlation Coefficient
CD4+	T-helper Lymphocytes (Cluster of Differentiation 4)
CEP	Research Ethics Committee
COM-HUPES	Professor Edgard Santos University Hospital Complex
CI	Confidence Interval
Ct	Cycle Threshold
DNA	Deoxyribonucleic Acid
DMSO	Dimethyl Sulfoxide
EDTA	Ethylenediaminetetraacetic Acid
FBS	Fetal Bovine Serum
g	Relative Centrifugal Force
HAM/TSP	HTLV-1-Associated Myelopathy/Tropical Spastic Paraparesis
HTLV-1	Human T-cell Lymphotropic Virus type 1
Log_10_	Decadic Logarithm (Base-10)
LoD	Limit of Detection
LoQ	Limit of Quantification
MGB-NFQ	Minor Groove Binder/Non-Fluorescent Quencher
NaCl	Sodium Chloride
PBMCs	Peripheral Blood Mononuclear Cells
PBS	Phosphate-Buffered Saline
Pol	HTLV-1 Polymerase Gene
PVL	Proviral Load
qPCR	Quantitative Polymerase Chain Reaction
QTL	Quantification Tamegão-Lopes
RPMI	Roswell Park Memorial Institute Medium
R2	Coefficient of Determination
SD	Standard Deviation
SOP	Standard Operating Procedure
µL	Microliter
WBV	Whole Blood Volume-Based Absolute Quantification
WHL	Whole Blood per 10^−3^ Leukocytes

## Appendix A. Raw HTLV-1 Proviral Load Quantification Data

This Appendix presents the raw quantification data for the 66 participants included in this study. The table below details the absolute number of HTLV-1 proviral copies across all the methodologies tested (Table A1). These values are presented before log_10_ transformation to allow for a direct assessment of analytical focus and the raw distribution of proviral load across different clinical profiles. All studies follow the methodological standards and descriptive correction factors described in the Materials and Methods Section of the main article.

**Table A1:** Individual values of HTLV-1 proviral load using different quantification methodologies for all study participants (n = 66).

Sample Code	Absolute Quantification (uL)	Tamegã-Lopes Method.	WB HTLV-1/1000	BC HTLV-1/1000	PBMCs HTLV-1/1000	CD4+ HTLV-1/1000
PRV-001	-	-	-	-	-	-
PRV-002	-	-	-	-	-	-
PRV-003	-	-	-	-	-	-
PRV-004	13	60	9		17	35
PRV-005	36	215	15		51	96
PRV-006	150	232	28		68	122
PRV-007	-	-	-	-	-	-
PRV-008	-	-	-	-	-	-
PRV-009	96	80	11		17	39
PRV-010	10	1	11	-	-	80
PRV-011	-	-	-	-	-	-
PRV-012	8	21	2	-	19	45
PRV-013	310	526	72	-	91	96
PRV-014	8	9	1	-	5	8
PRV-015	-	-	-	-	-	-
PRV-016	-	-	-	-	-	-
PRV-017	-	-	-	-	-	-
PRV-018	160	305	47	-	-	126
PRV-019	131	247	31	-	64	171
PRV-020	54	112	8	-	-	25
PRV-021	98	129	17	-	-	0
PRV-022	4	18	2	-	-	6
PRV-023	142	163	22	11	-	-
PRV-024	16	48	5	4	-	17
PRV-025	127	2571	223	22	-	21
PRV-026	15	32	3	3	-	18
PRV-027	554	704	46	50	200	199
PRV-028	212	544	70	53	138	239
PRV-029	169	197	17	19	40	91
PRV-030	85	311	49	30	61	33
PRV-031	192	495	51	34	99	128
PRV-032	376	584	64	64	100	150
PRV-033	28	48	3	4	10	18
PRV-034	65	134	15	14	31	15
PRV-035	65	103	8	9	21	36
PRV-036	82	88	9	10	20	20
PRV-037	45	47	4	4	18	52
PRV-038	3	4	0	1	2	3
PRV-039	16	31	4	4	21	30
PRV-040	-	-	-	-	-	-
PRV-041	122	237	14	13	32	50
PRV-042	211	531	64	61	170	550
PRV-043	14	17	2	2	4	14
PRV-044	66	85	10	11	16	42
PRV-045	5	5	-	-	2	3
PRV-046	143	312	23	21	62	87
PRV-047	147	822	69	64	95	234
PRV-048	57	293	32	27	59	110
PRV-049	68	415	33	22	63	103
PRV-050	2	22	2	1	2	6
PRV-051	369	466	20	28	29	203
PRV-052	256	268	18	28	34	89
PRV-053	71	87	4	4	-	41
PRV-054	148	203	39	43	52	141
PRV-055	-	-	-	-	-	-
PRV-056	330	726	45	30	115	222
PRV-057	6277	7984	383	399	552	682
PRV-058	1	2	-	-	31	1
PRV-059	369	508	33	35	117	278
PRV-060	4	4	1	1	-	2
PRV-061	300	294	16	50	39	22
PRV-062	-	-	-	-	-	-
PRV-063	16	21	3	3	136	7
PRV-064	82	127	14	4	27	34
PRV-065	31	44	6	15	12	37
PRV-066	13	17	3	3	9	9

Note: All PVL values are expressed in absolute numbers (copies/µL or copies/1000 cells), as indicated in each column. Abbreviations: WB, whole blood; BC, buffy coat; PBMCs, peripheral blood mononuclear cells; CD4+, isolated CD4+ T cells; HTLV-1, human T-cell lymphotropic virus type 1. Missing data: Cells marked with “-” indicate samples that did not reach the detection limit or were unavailable for that specific matrix.

## Appendix B. Analytical Performance and Validation of the qPCR Assays

This Appendix details the experimental validation of qPCR assays for determining the standard curve for quantifying the proviral load of HTLV-1 and the reference gene (ALB).

### Appendix B.1. Assay Sensitivity and Dynamic Range

Table A2 summarizes the analytical validation of the qPCR standard curves. The standard material containing the HTLV-1 and ALB targets was purified and quantified using a Qubit fluorimetric assay to establish the initial concentration. A series of 10-fold serial dilutions (ranging from 10^−1^ to 10^−14^) were prepared and tested in duplicate, allowing the determination of the mean Ct values. This process covered the linear dynamic range of the assay and provided the basis for the regression parameters used in the quantification of the absolute proviral load.

**Table A2:** Mean qPCR cycle threshold values in serial dilutions.

Standard Dilution	HTLV-1 Target Ct (Mean)	ALB Target Ct (Mean)
10^−1^	60.50 *	60.50 *
10^−2^	4.53	5.52
10^−3^	9.39	10.42
10^−4^	13.03	13.28
10^−5^	16.06	16.51
10^−6^	19.97	20.24
10^−7^	23.29	23.94
10^−8^	26.40	27.22
10^−9^	30.52	30.33
10^−10^	33.47	33.70
10^−11^	36.55	37.55
10^−12^	37.91	60.50 *
10^−13^	60.50 *	60.50 *
10^−14^	60.50 *	60.50 *

Amplification Status (*): Values 60.00 and 61.00 were assigned to samples that did not amplify within the standard cycle of 40 repetitions.

Establishing the theoretical number of DNA copies was essential for constructing standard curves for the HTLV-1 and ALB targets. By correlating the mean cycle threshold (Ct) values with the base-10 logarithm (log_10_) of these theoretical copies, a linear regression model was developed to allow for WBV (copies/µL of blood). Initial dilutions (10^−1^ and 10^−2^) were excluded from the regression analysis due to PCR inhibition caused by the high template concentration. Similarly, dilutions above 10^−11^ were omitted as they reached the stochastic limit of the assay, where the low DNA concentration leads to inconsistent amplification or results below the limit of quantification (LoQ).

### Appendix B.2. Standard Curve Analysis

Figure A1 presents the standard curves used for the WBV (copies/µL of blood) of the HTLV-1 proviral load. To establish this model, a correlation was performed between the mean cycle threshold (Ct) values and the base-10 logarithm (log_10_) of the theoretical number of DNA copies obtained through serial dilutions. HTLV-1 Target: The standard curve for the pol gene (Figure A1a) demonstrated high linearity (R^2^ = 0.998) and an amplification efficiency of 94.5%, with a slope of −3.46. Endogenous Control: The albumin (ALB) curve (Figure A1b) showed a slope of −3.38 and an efficiency of 97.6% (R^2^ = 0.998), ensuring accurate cellular normalization for all clinical samples (Table A4). The graphs illustrate the reliable linear dynamic range of the assay; points representing dilutions that did not amplify or showed inhibition (as detailed in Table A3 and Table A4) were excluded from the regression to maintain the accuracy of the quantification parameters.

**Table A3:** Theoretical DNA copy numbers and *log*_10_ values for qPCR standard curves, expressed in absolute copies per µL.

Standard Dilution	HTLV-1 Copies		Albumin Copies	
	Copies/µL	LOG_10_	Copies/µL	LOG_10_
*^a^* 10^−1^	6,075,692,075	9.78	9,337,945,287	9.97
*^a^* 10^−2^	607,569,207	8.78	933,794,529	8.97
10^−3^	60,756,921	7.78	93,379,453	7.97
10^−4^	6,075,692	6.78	9,337,945	6.97
10^−5^	607,569	5.78	933,795	5.97
10^−6^	60,757	4.78	93,379	4.97
10^−7^	6076	3.78	9338	3.97
10^−8^	608	2.78	934	2.97
10^−9^	61	1.78	93	1.97
10^−10^	6	0.78	9	0.97
*^b^* 10^−11^	1	0*	1	0*
*^b^* 10^−12^	0*	0*	0*	0*
*^b^* 10^−13^	0*	0*	0*	0*
*^b^* 10^−14^	0*	0*	0*	0*

Logarithmic Scale: The log_10_ transformation of the copy numbers was employed to establish a linear relationship with the cycle threshold (Ct) values during regression analysis. “*a*” Initial dilutions (10^−1^–10^−2^): Excluded from the linear regression analysis due to observed PCR inhibition caused by high template concentration. “*b*” Limit of Detection: Dilutions beyond (10^−11^) were excluded from the standard curve as they fell outside the validated linear dynamic range or reached the assay’s stochastic limit. Dynamic Range: Values for dilutions from 10^−12^ to 10^−14^ are represented as “0*” in this table to indicate that they are below the limit of quantification (LoQ) calculated by the assay.

**Table A4:** Regression parameters and amplification efficiency for HTLV-1 and ALB standard curves.

Standard Curve	HTLV-1	ALB
*a*	−0.288	−0.295
*b*	10.499	10.953
Slope	−3.461	−3.386
Efficiency (%)	94.493	97.379
*R* ^2^	0.998	0.998

Regression Equation: The parameters follow the standard linear model *Y* = *aX* + *b*, where *Y* is the cycle threshold (Ct) and *X* is the log_10_ of the DNA copy number. Intercept (*b*): Represents the theoretical Ct value for a single copy of the target gene. Efficiency (%): Calculated using the formula *E* = (−1 + 10^−1/slope^) × 100.

### Appendix B.3. Determining the Correction Factor

The original eluate was diluted 1:10 in physiological saline solution and then subjected to a second extraction process (re-extraction) to measure the efficiency of DNA recovery compared to the primary eluate (original extract). The “Difference Ct” was calculated by subtracting the re-extraction Ct from the Ct of the original extract for each paired sample. Correction Factor: An average correction factor of 2.01 Cts was derived from the observed differences (Table A5). This value was mathematically integrated into the final quantification formula to normalize the results and account for DNA loss during the extraction process.

**Table A5:** Determination of the extraction correction factor for proviral load normalization.

Original Extract Ct	Re-Extract Ct	Difference Ct
27.89	25.75	2.14
28.05	25.84	2.21
28.19	26.09	2.10
34.42	32.46	1.96
34.46	32.57	1.89
34.58	32.84	1.74
Mean = Correction Factor =		2.01

### Appendix B.4. Sensitivity Analysis of Correction Factor Magnitude

Sensitivity analyses were performed to evaluate the impact of the magnitude of the correction factor on the agreement between WBV (copies/µL of blood) and QTL method [19]. Additional analyses were conducted using three alternative scenarios: no correction factor, minimum correction factor and maximum correction factor (Table A6).

**Table A6:** Sensitivity analysis of extraction correction-factor scenarios for proviral load normalization.

Correction-Factor Scenario	ΔCt	*r*	Mean Bias	CCC (95%)
With Correction Factor	2.01	0.928	−0.273	0.866 (0.793–0.915)
Without Correction Factor	0	0.926	+0.288	0.855 (0.776–0.907)
Minimum Correction Factor	1.74	0.926	−0.214	0.885 (0.817–0.929)
Maximum Correction Factor	2.21	0.926	−0.350	0.825 (0.738–0.885)

Correlation, concordance, and agreement metrics between absolute quantification and Tamegão-Lopes method across different correction-factor scenarios. Analyses include no correction, minimum, and maximum ΔCt-based correction factors. Pearson correlation coefficient (*r*), mean bias from Bland–Altman analysis, and Lin’s concordance correlation coefficient (CCC) with 95% confidence intervals are presented.
