# Correction: Franco et al. Whole Blood Volume-Based Absolute Quantification of HTLV-1 Proviral Load: A Comparative Method Evaluation Study. *Viruses* 2026, *18*, 580

**DOI:** 10.3390/v18060663

**Published:** 2026-06-12

**Authors:** Gabriel O. Franco, Andreas Stocker, Eduardo M. Netto, Heliene Pereira, Carlos Brites

**Affiliations:** 1 Laboratório de Pesquisa em Infectologia (LAPI), Complexo Hospitalar Universitário Professor Edgard Santos, Salvador 40110-060, Brazil; gabriel.osf22@gmail.com (G.O.F.); astocker@ufba.br (A.S.); nettoeduardom@hotmail.com (E.M.N.); helienepereira@outlook.com (H.P.); 2 Programa de Pós-Graduação em Medicina e Saúde (PPgMS), Universidade Federal da Bahia (UFBA), Salvador 40110-060, Brazil; 3 Centro de Formação em Biologia Molecular Charles Merieux (CFBMCM), Professor Edgard Santos University Hospital Complex, Salvador 40110-060, Brazil

## Text Correction

In the original publication [1], the description of the procedure used to establish the extraction correction factor incorrectly stated that the eluates were diluted prior to re-extraction. The corrected description is as follows:

2.7. Standard Curve

A correction factor was established to ensure result reliability and compensate for systematic variations associated with the extraction process. Six samples were initially extracted and subjected to a new extraction step. qPCR analysis showed a mean difference of ΔCt = 2.01 between the original and re-extracted eluates. Considering the exponential nature of qPCR, this difference corresponds to approximately a four-fold variation in copy number estimation. This ΔCt value was incorporated as a correction factor in the final equations for all HTLV-1 PVL calculations and was added to the Ct values obtained from clinical samples prior to conversion into copy numbers. The application of this correction factor was standardized to reduce variability associated with the extraction process.

Appendix B.3. Determining the Correction Factor

The original eluate was subjected to a second extraction process (re-extraction) to measure the efficiency of DNA recovery compared to the primary eluate (original extract). The “Difference Ct” was calculated by subtracting the re-extraction Ct from the Ct of the original extract for each paired sample. Correction Factor: An average correction factor of 2.01 Cts was derived from the observed differences (Table A5). This value was mathematically integrated into the final quantification formula to normalize the results and account for DNA loss during the extraction process.

The authors state that the scientific conclusions are unaffected. This correction was approved by the Academic Editor. The original publication has also been updated.
